# Acute Effusive Pericarditis: A Late Complication of COVID-19

**DOI:** 10.7759/cureus.9074

**Published:** 2020-07-08

**Authors:** Kelli Fox, Jessica A Prokup, Kyle Butson, Kim Jordan

**Affiliations:** 1 Internal Medicine, OhioHealth Riverside Methodist Hospital, Columbus, USA

**Keywords:** covid-19, pericarditis, pericardial effusion, tamponade, sars-cov-2

## Abstract

As the COVID-19 pandemic evolves, the medical community continues to discover novel clinical manifestations of the severe acute respiratory syndrome coronavirus 2 (SARS-CoV-2) virus. Often, extrapulmonary manifestations occur simultaneously with pulmonary disease. However, there is a paucity of literature describing the cardiac manifestations of COVID-19 in the absence of pulmonary findings. We report a rare case of acute pericarditis presenting with pericardial effusion and cardiac tamponade in a 43-year-old man diagnosed with COVID-19. This case emphasizes the importance of continued investigation regarding diagnosis and treatment of COVID-19 and its related symptoms.

## Introduction

Several reports have described pulmonary involvement as the most common clinical presentation of severe infection with the severe acute respiratory syndrome coronavirus 2 (SARS-CoV-2) virus (COVID-19) [1-4]. As the outbreak progressed to a global pandemic, extrapulmonary manifestations were increasingly identified, most often occurring concomitantly with pulmonary disease [5-6]. Pericardial disease in the absence of pulmonary findings, however, is rarely described [7-9]. We report acute pericarditis presenting with pericardial effusion and cardiac tamponade in an adult man with COVID-19.

## Case presentation

A 43-year-old African American man with no past medical history presented to the emergency department with a four-day history of progressive orthopnea, conversational dyspnea, and chest pain radiating to the neck and left shoulder. He reported a mild non-productive cough and subjective fever two weeks prior to presentation but had no other associated symptoms. He denied known sick contacts, recent travel (including international), and took no home medications. The patient is a native of East Africa but lived in the United States for the past 13 years. He denied a personal history of tuberculosis (TB) or any known TB contacts, personal or family history of autoimmune conditions, or personal history of joint pain or swelling.

**Figure 1:**
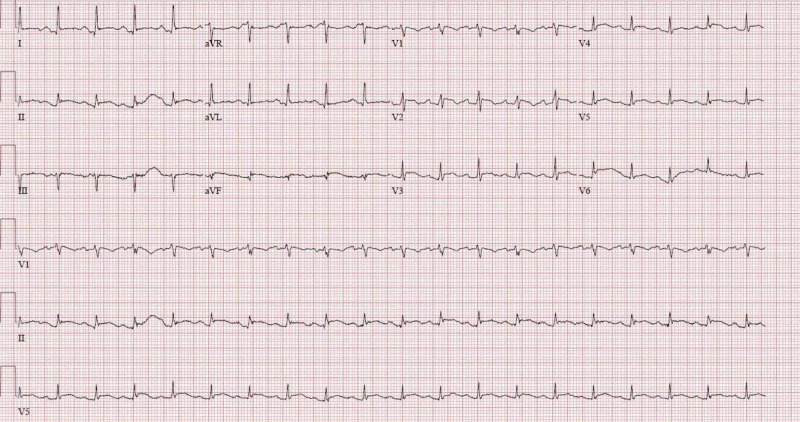
Electrocardiogram (EKG) 12-lead EKG demonstrates sinus tachycardia with diffuse concave ST-segment elevation as well as PR-segment depression, except in aVR showing PR elevation, consistent with acute pericarditis.

En route to the emergency department, telemetry was interpreted as ST-elevation myocardial infarction (STEMI). Emergency medical services subsequently administered 324 mg aspirin, heparin 5000 units, and nitroglycerin paste. On arrival, he was tachycardic and tachypneic with a blood pressure of 142/96 mmHg. Initial oxygen saturation was 98% on 4 liters nasal cannula and the patient was weaned successfully to 2 liters nasal cannula for symptomatic relief. Physical examination showed elevated jugular venous pressure and pulsus paradoxus on inspiration. Cardiopulmonary examination was significant for muffled heart sounds, friction rub, and bilateral rhonchi. A 12-lead electrocardiogram revealed sinus tachycardia with low voltage as well as diffuse concave ST-elevation and PR-segment depression, with PR-elevation in aVR (Figure 1).

**Figure 2:**
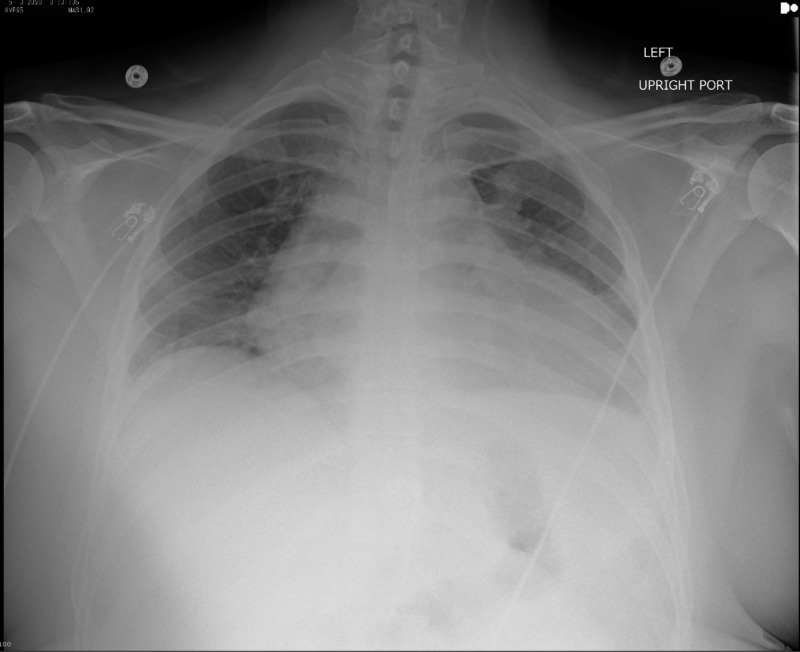
Chest X-ray Chest X-ray demonstrates cardiomegaly with the absence of pulmonary infiltrates.

His clinical features were concerning for COVID-19, and testing via nasopharyngeal molecular polymerase chain reaction (PCR) for SARS-CoV-2 yielded a positive result. Chest X-ray was significant for cardiomegaly with the absence of pulmonary infiltrates (Figure 2).

A point of care ultrasound revealed a large pericardial effusion with concern for tamponade physiology. He was admitted to the ICU for close hemodynamic monitoring and special isolation with negative airflow.

**Figure 3:**
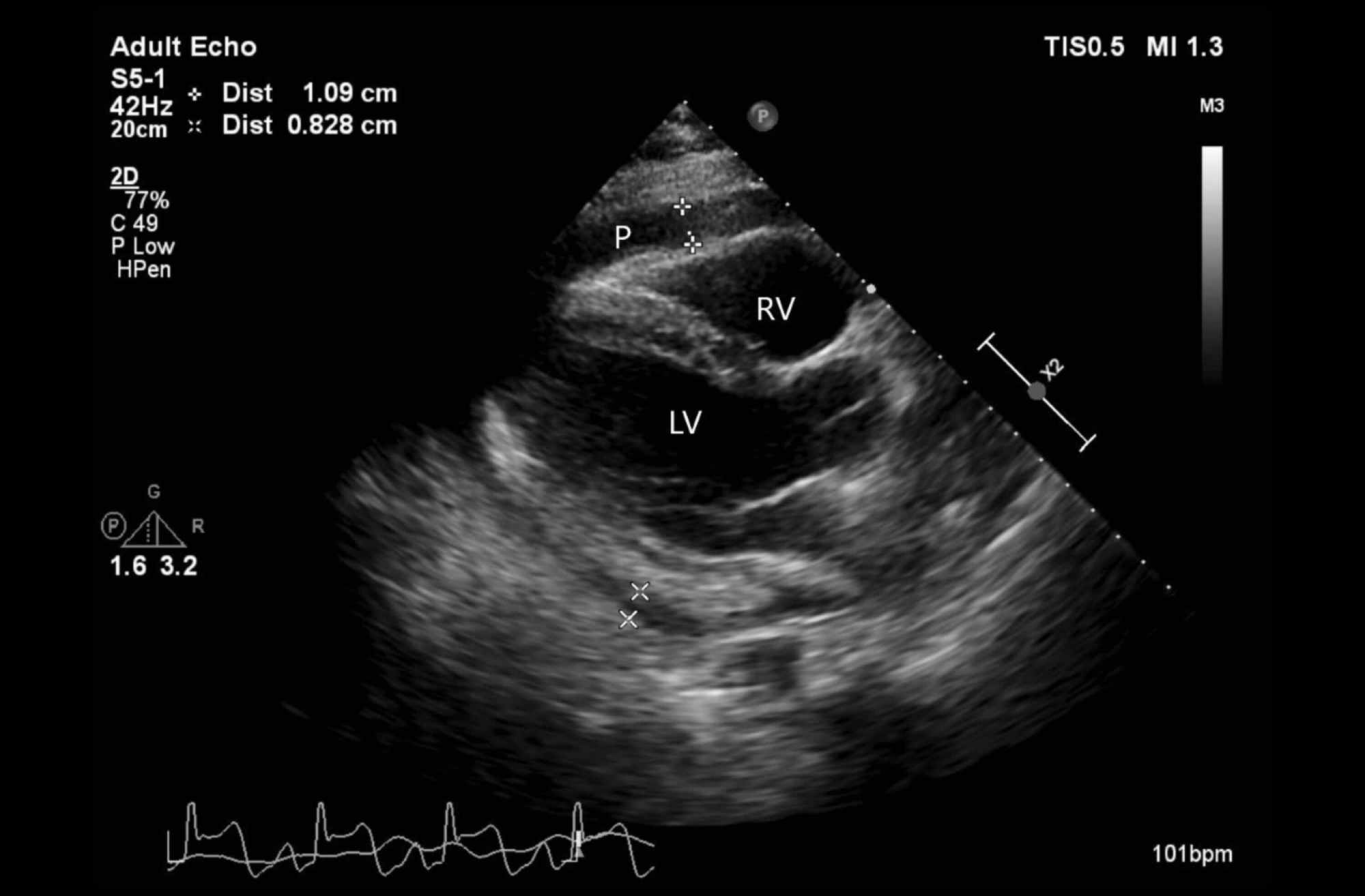
Parasternal long axis of transthoracic echocardiogram (TTE) Parasternal long axis view of TTE demonstrates circumferential effusion in pericardial space (P). LV, left ventricle. RV, right ventricle.

**Figure 4:**
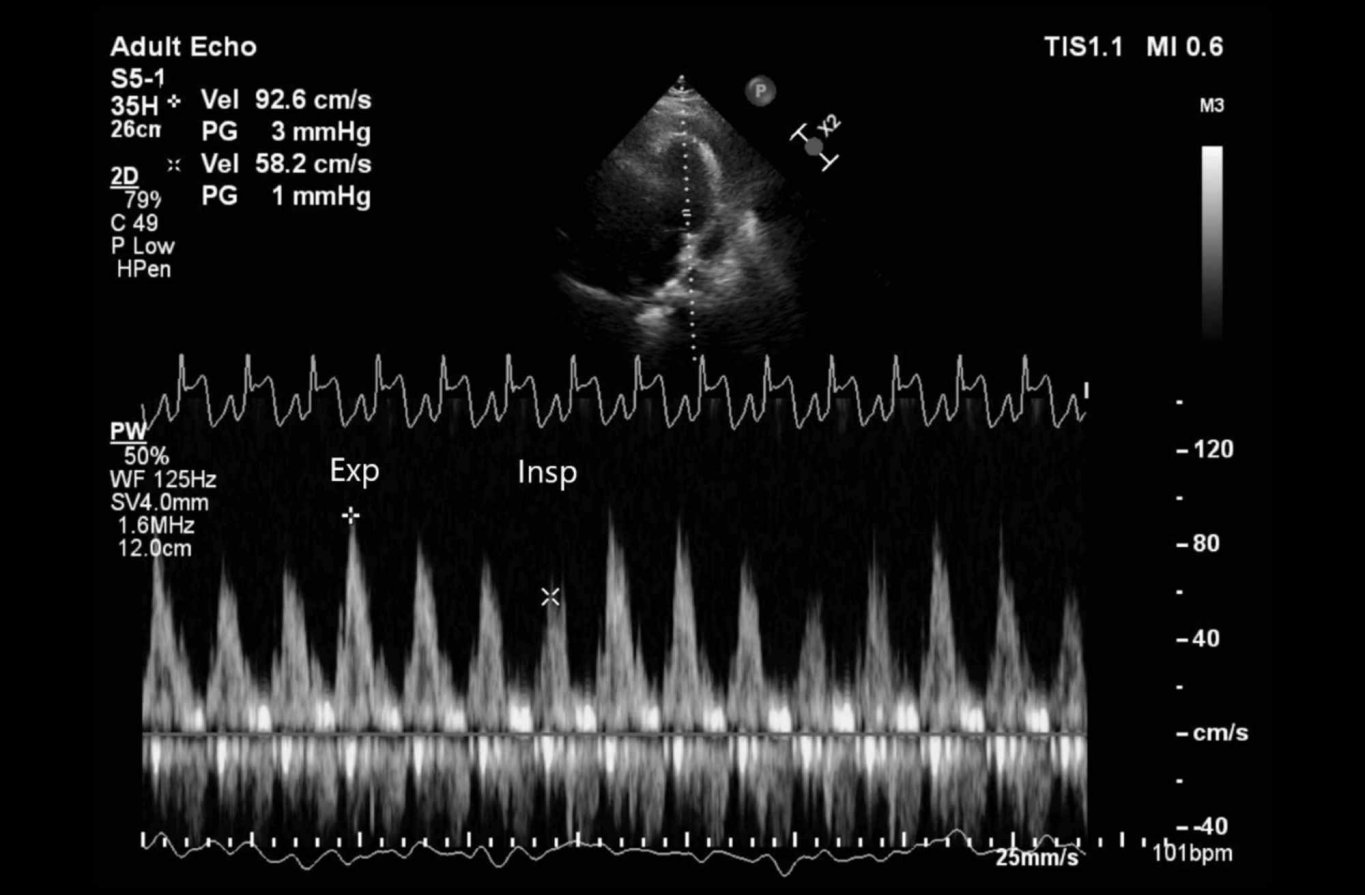
Pulse-wave Doppler of left ventricular inflow on transthoracic echocardiogram (TTE) Pulse-wave Doppler of left ventricular inflow at mitral valve level on TTE demonstrates prominent inspiratory reduction in flow (Insp) compared to expiration (Exp), consistent with tamponade physiology.

Initial laboratory studies revealed leukocytosis white blood cells (WBC) 12.17 K/mcL (4.5-11 K/mcL) without lymphopenia, thrombocytosis 610 K/mcL (150-400 K/mcL), and elevated inflammatory markers including D-dimer 6.32 mcg/mL fibrinogen equivalent units (FEU) (<0.5 mcg/mL FEU), ferritin 1077 ng/mL (30-400 ng/mL), lactate dehydrogenase (LDH) 623 U/L (100-250 U/L), C-reactive protein (CRP) 368 mg/L (0-10 mg/L), antinuclear antibodies (ANA) 1:320 with homogeneous pattern (<80 titer), and rheumatoid factor 15.5 IU/mL (0-14 IU/mL). Two high sensitivity cardiac troponin assays were drawn three hours apart and were both <6 ng/L (<22 ng/L). Procalcitonin, creatine phosphokinase (CPK), and lactic acid levels were within normal limits. Testing for human immunodeficiency virus (HIV) antibodies and T-Spot TB were negative. A comprehensive transthoracic echocardiogram confirmed a moderate circumferential pericardial effusion with respiratory variation to left ventricular inflow, suggestive of early tamponade physiology (Figures 3-4).

**Figure 5:**
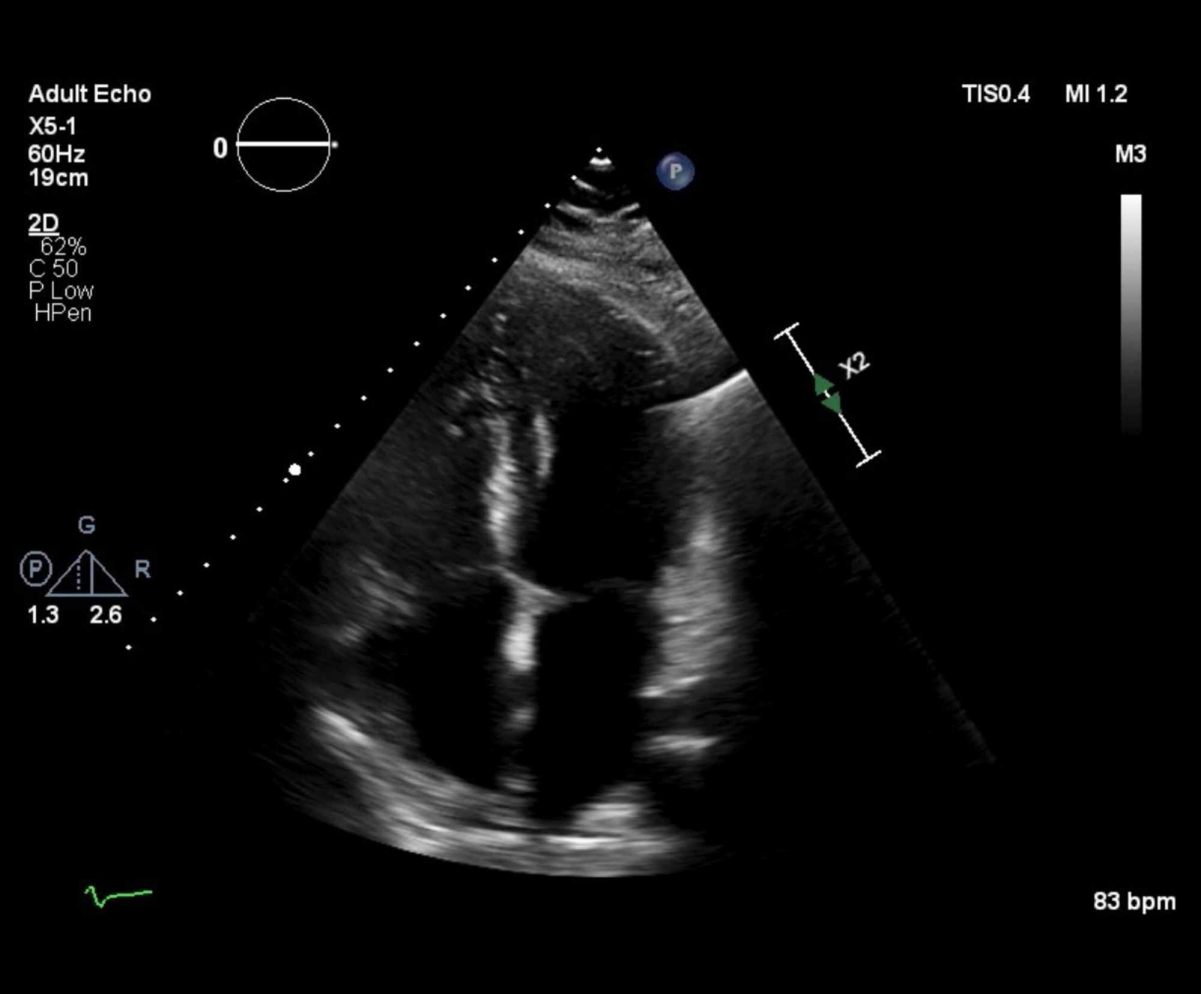
Apical four chamber view at one month post discharge Apical four chamber view on transthoracic echocardiogram (TTE) demonstrates resolution of pericardial effusion one month following discharge.

**Figure 6:**
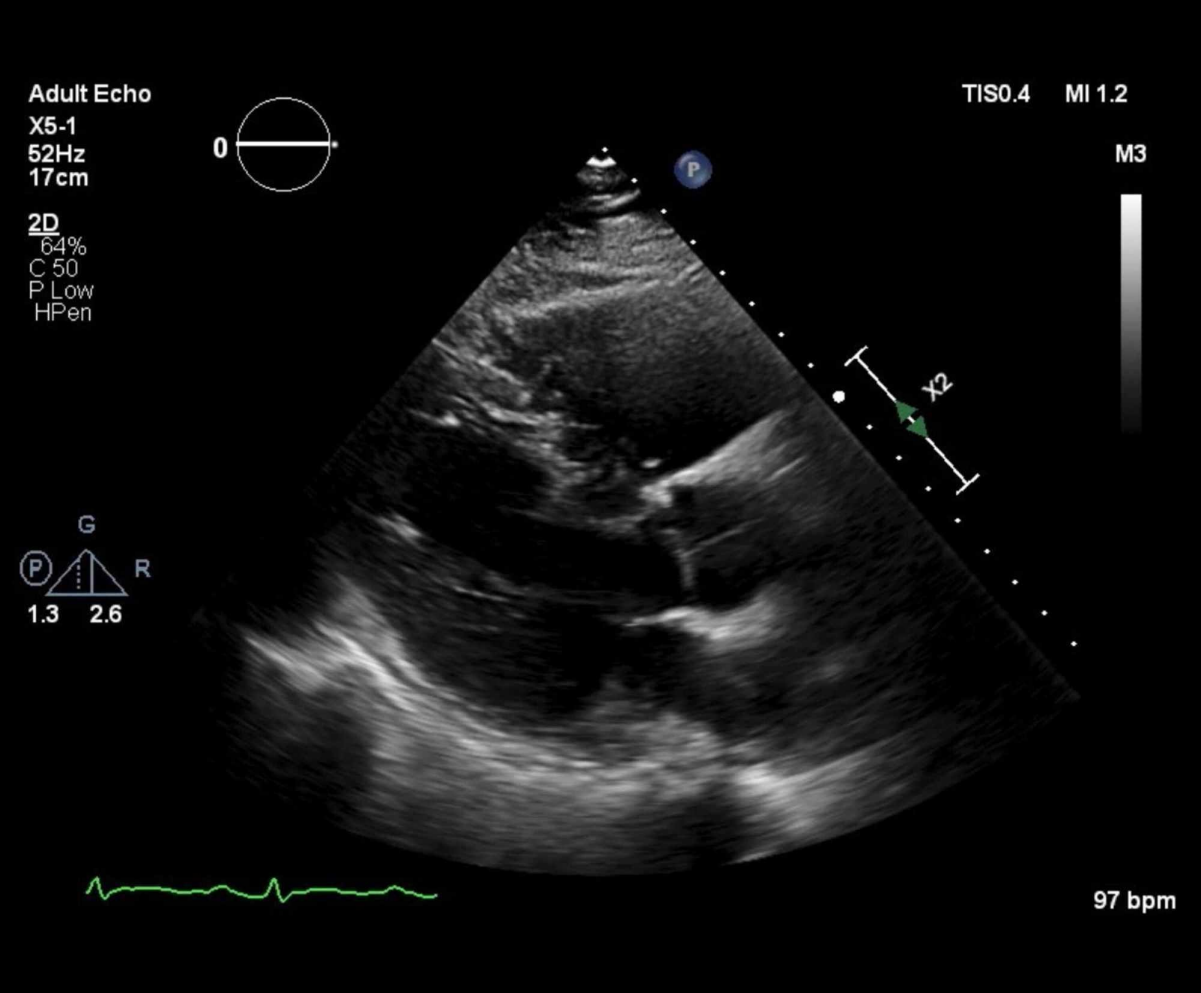
Parasternal long axis view on transthoracic echocardiogram (TTE) at one month post discharge Parasternal long axis view of TTE demonstrates resolution of pericardial effusion one month following discharge.

He underwent pericardiocentesis with the removal of 400 mL of serosanguinous fluid. Over the next 24 hours, the patient’s pericardial drain produced an additional 525 mL of serosanguinous fluid. He was initiated on colchicine and ibuprofen therapy for pericarditis with improvement in his symptoms. Pericardial fluid analysis revealed red blood cells (RBC) 50,3775/mcL, WBC >50,000/mcL with neutrophil predominance, and LDH 2270 U/L. Cytology and fungal, aerobic, and anaerobic cultures of the pericardial fluid were negative. Molecular testing performed on the pericardial fluid was negative for COVID-19. During his hospital course, his leukocytosis resolved, CRP levels decreased, and chest pain significantly improved. He was discharged home in stable condition with colchicine 0.6 mg two times daily for three months and ibuprofen 600 mg three times daily with plans for a gradual taper following complete resolution of symptoms and CRP normalization. One month following discharge, repeat echocardiogram showed complete resolution of his pericardial effusion (Figures 5-6).

## Discussion

There is a paucity of literature describing the cardiac manifestations of COVID-19. Dabbagh et al. reported a 67-year-old woman who tested positive for SARS-CoV-2 one week prior to presentation with a large hemorrhagic pericardial effusion complicated by tamponade and Takotsubo cardiomyopathy [7]. A second case described a previously healthy 53-year-old Caucasian woman who tested positive for SARS-CoV-2 and presented with acute myopericarditis with circumferential pericardial effusion and left ventricular systolic dysfunction one week after the development of COVID-19 symptoms [8]. Another case involved a 70-year-old West African woman who developed COVID-19-related pericarditis two weeks following treating for a non-ST-elevation myocardial infarction (NSTEMI) [9]. Similar to our patient, there was no evidence of interstitial pulmonary disease or pneumonia in these patients. However, in comparison, our patient’s case showed no evidence of myocardial inflammation, myocardial injury, or underlying structural abnormality.

Although the pathophysiology of effusive pericarditis in COVID-19 is unknown, it is hypothesized that it occurs secondary to the systemic inflammatory response and the subsequent cytotoxic and immune-mediated effects related to SARS-CoV-2 [7-8,10]. Importantly, inflammatory markers and autoantibody testing are frequently abnormal in the setting of acute infection and often require clinical findings for the determination of underlying etiology [11]. Pericardial effusion and tamponade secondary to COVID-19 infection remain a plausible explanation for our patient’s findings given the absence of other etiologic findings, the temporal relationship to his preceding illness, and positive COVID-19 testing. Unfortunately, a validated test to assess COVID-19 in the pericardial fluid has yet to be developed. For academic purposes, our institution ran a COVID-19 molecular PCR test, intended for nasopharyngeal specimens, on a sample of the patient’s pericardial fluid, which yielded a negative result. To date, COVID-19 molecular PCR testing of pericardial fluid has yet to yield a positive result, which is not unlike other viruses as detection in the pericardial fluid is often difficult with low overall yield [5]. Further studies are needed to determine whether COVID-19 can survive in pericardial fluid and how to accurately test for its presence.

Currently, there are no established guidelines for the management of pericarditis secondary to COVID-19. In the current reported cases, patients were treated with colchicine, hydroxychloroquine, steroids, and antivirals [7-9]. Our patient had a rapid clinical response to standard therapy (ibuprofen and colchicine) for viral pericarditis, thus we did not consider additional treatments. Future work is needed to determine whether standard treatment alone or standard treatment plus antiviral therapy yields superior outcomes in COVID-19-related pericarditis.

## Conclusions

Acute effusive pericarditis is a rare manifestation of COVID-19, especially without concomitant pulmonary disease or myocardial injury. It is important to maintain a high level of suspicion in symptomatic patients in order to assure early diagnosis and treatment. Future studies are needed to determine how to accurately test for the presence of COVID-19 in pericardial fluid as well as to determine a preferred treatment for these patients.

## References

[1] Lin C, Chen Z, Xie B (2020). COVID-19 pneumonia patient without clear epidemiological history outside Wuhan: an analysis of the radiographic and clinical features. Clin Imaging.

[2] Konopka KE, Wilson A, Myers JL (2020). Postmortem lung findings in an asthmatic patient with coronavirus disease 2019. Chest.

[3] Ahmed T, Shah RJ, Rahim SEG, Flores M, Olinn A (2020). Coronavirus disease 2019 (COVID-19) complicated by acute respiratory distress syndrome: an internist’s perspective. Cureus.

[4] Schuster J, Spinner CD, Schuldt A (2020). A moderate case of COVID-19 viral pneumonia during the SARS-CoV-2 pandemic. Dtsch Arztebl Int.

[5] Hua A, O’Gallagher K, Sado D, Byrne J (2020). Life-threatening cardiac tamponade complicating myo-pericarditis in COVID-19. Eur Heart J.

[6] Cizgici AY, Agus HZ, Yildiz M (2020). COVID-19 myopericarditis: it should be kept in mind in today's conditions. Am J Emerg Med.

[7] Dabbagh MF, Aurora L, D’Souza P, Weinmann AJ, Bhargava P, Basir MB (2020). Cardiac tamponade secondary to COVID-19 [Epub ahead of print]. J Am Coll Cardiol.

[8] Inciardi RM, Lupi L, Zaccone G (2020). Cardiac involvement in a patient with coronavirus disease 2019 (COVID-19). JAMA Cardiol.

[9] Asif T, Kassab K, Iskander F, Alyousef T (2020). Acute pericarditis and cardiac tamponade in a patient with COVID- 19: a therapeutic challenge. Eur J Case Rep Intern Med.

[10] Adler Y, Charron P, Imazio M (2015). 2015 ESC Guidelines for the diagnosis and management of pericardial diseases: the task force for the diagnosis and management of pericardial diseases of the European Society of Cardiology (ESC). Eur Heart J.

[11] Litwin CM, Binder SR (2016). ANA testing in the presence of acute and chronic infections. J Immunoassay Immunochem.

